# Métastase pariétale d’un adénocarcinome bronchique

**DOI:** 10.11604/pamj.2019.32.100.14373

**Published:** 2019-03-04

**Authors:** Yasser El Brahmi, Mbarek Yakka, Abdelkader Ehirchiou, Mountassir Moujahid, Aziz Zentar

**Affiliations:** 1 Service de Chirurgie Viscérale 2, Hôpital Militaire d’Instruction Mohammed V, Rabat, Maroc

**Keywords:** Tumeur bronchique, métastase cutanée, chirurgie palliative, Bronchial tumor, skin metastasis, palliative surgery

## Abstract

Les métastases cutanées du carcinome bronchogénique sont rares, elles témoignent d'un stade tardif de la maladie. Nous rapportons le cas d'une métastase cutanée d'un adénocarcinome bronchique traitée chirurgicalement en vue d'améliorer le confort du malade.

## Abstract (en)

Skin metastases from bronchogenic carcinoma are rare, indicating advanced disease. We here report a case of skin metastasis from bronchial adenocarcinoma treated surgically in order to improve patient comfort.

## Introduction

Nous rapportons le cas d'une métastase cutanée d'un cancer bronchique sous forme d'une masse pariétale en regard de la fosse iliaque droite, avec une revue de la littérature.

## Patient et observation

**Figure 1:**
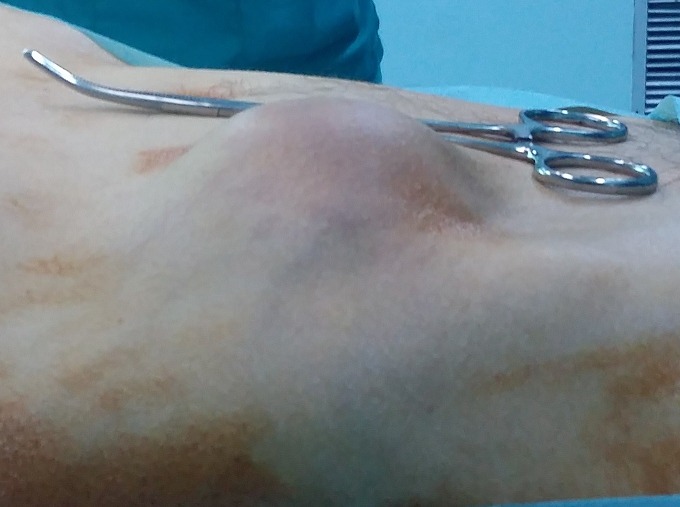
image préopératoire de la masse pariétale

**Figure 2:**
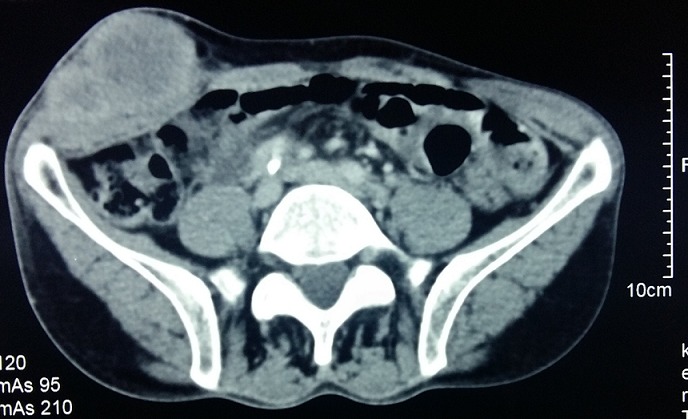
image scannographique de la masse pariétale

**Figure 3:**
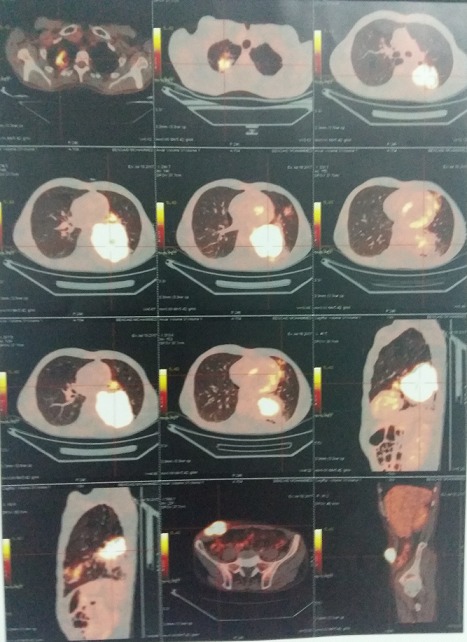
signes d’hyper métabolismes au pet-scan

**Figure 4:**
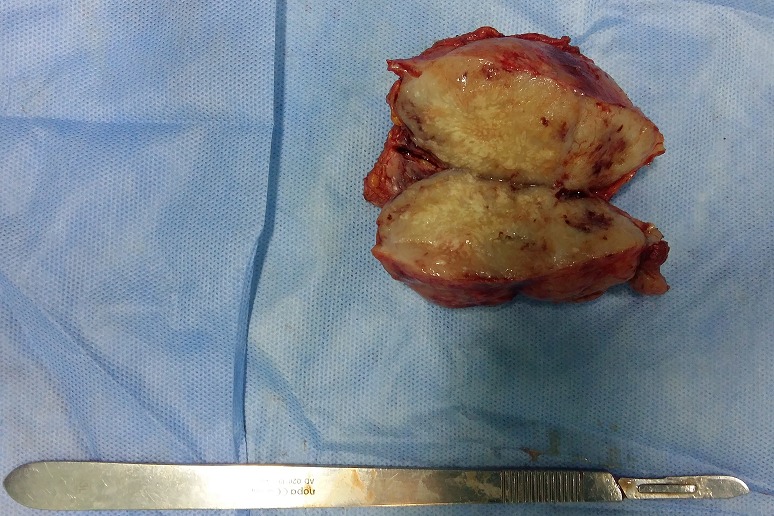
pièce opératoire

Il s'agit d'un patient âgé de 60 ans, ayant comme antécédents un tabagisme chronique à raison de 40 paquets-années et une tuberculose pulmonaire traité et déclaré guérie en 1975. Le patient était suivi au service de pneumologie pour un adénocarcinome bronchique au stade métastatique pour lequel il a bénéficié d'une première cure de chimiothérapie puis transféré au service de chirurgie viscérale suite à l'apparition d'une masse pariétale en regard de la fosse iliaque droite qui augmentait rapidement de taille et qui est très douloureuse à la palpation avec des signes d'inflammation en regard (Figure 1). Une TDM abdominale objectivait une masse tissulaire du flanc droit homogène polylobée mesurant 70mm*36mm*55 mm sans extension intra abdominal (Figure 2). Le pet-scan montrait une masse sous cutanée de la fosse iliaque droite bilobée, SUV max = 12,2 mesurant 56mm*35mm*49mm (Figure 3). Vu le caractère douloureux et expansive de la masse pariétale, on a décidé de la reséquer dans sa totalité en emportant la partie cutanée en regard (Figure 4). La pièce est adressée pour un examen anatomopathologique. L'étude anatomopathologique concluait à une lésion secondaire d'origine bronchique; il s'agissait d'un processus adénocarcinomateux moyennement différencié. Les suites étaient simples, il a quitté le service au troisième jour. Le patient a repris sa deuxième cure de chimiothérapie vingt jours plus tard, malheureusement il est décédé un mois après l'intervention chirurgicale.

## Discussion

Les métastases cutanées sont rares et leur fréquence est estimée à 4% dans les cancers viscéraux [1]. Chez l'homme 25% des métastases cutanées sont d'origine pulmonaire [2]. La formation des métastases cutanées se fait le plus souvent par voie lymphatique, ou hématogène [3]. La représentation clinique est souvent sous la forme de nodules dermiques ou hypodermiques de taille variable et de nombre en général limitée, de croissance rapide, finissant par se stabiliser dans leur expansion. La peau en regard est normale ou inflammatoire; voire même ulcérée [4]. Les métastases cutanées sont typiquement indolores lorsqu'elles ne sont ni volumineuses ni infectées. Le diagnostic se fait soit par la réalisation d'une biopsie ou par examen anatomopathologique de la pièce chirurgicale [5]. Le traitement symptomatique des métastases cutanées dépend surtout du cancer d'origine. Les métastases cutanées n'étant pas toujours douloureuses lors de leur apparition, il ne sera pas nécessaire de les traiter. Des métastases cutanées extensives et en rapide croissance peuvent par contre provoquer des douleurs importantes, un traitement palliatif de confort pourra alors être proposé [6]. Dans le cas présenté, notre patient se plaignait des douleurs atroces au niveau de la fosse iliaque droite avec augmentation manifeste de sa taille et impotence fonctionnelle; ce qui a conduit à réaliser une intervention chirurgicale pour ablation de cette masse. Le pronostic reste très péjoratif.

## Conclusion

Les métastases cutanées sont un signe de mauvais pronostic, et surviennent le plus souvent à un stade tardif au cours du développement de la pathologie tumorale. La bonne prise en charge thérapeutique et le suivi régulier des patients pourraient aider à réduire la fréquence des métastases cutanées.

## Conflits d’intérêts

Les auteurs ne déclarent aucun conflit d'intérêts.

## References

[1] Lookingbill DP, Spangler N, Helm KF (1993). Cutaneous metastases in patients with metastatic carcinoma: a retrospective study of 4020 patients. J Am Acad Dermatol.

[2] Rousseau A, Madinier JF, Favre A, Michenet P (1992). Tumeurs métastatiques des parties molles de la main. Ann Chir Main.

[3] Barnhill RL, Lugassy C (2004). Angiotropic malignant melanoma and extravascular migratory metastasis: description of 36 cases with emphasis on a new mechanism of tumour spread. Pathology.

[4] Rose BA, Wood FM (1983). Metastatic bronchogenic carcinoma masquerading as a felon. J Hand Surg Am.

[5] Horn Gary F (1996). À propos d'un cas de métastase d'un carcinome épidermoïde au niveau de la pulpe digitale. Ann Chir Plast Esth.

[6] Oualla K, Arifi S, Mellas N, El Mesbahi O (2012). Cutaneous metastases of internal cancers: a retrospective study about 12 Cases. J Cancer Sci Ther.

